# Patient Reported Pain and Disability Following a Distal Radius Fracture: A Prospective Study

**DOI:** 10.2174/1874325001711010589

**Published:** 2017-07-31

**Authors:** Emily Lalone, Joy MacDermid, Ruby Grewal, Graham King

**Affiliations:** 1 Western Univeristy - Mechanical and Materials Engineering, 1151 Richmond Street, London, Ontario N6A 5B9, Canada; 2 McMaster University - School of Rehabilitation Science, Rm 429, IAHS Victoria, Hamilton, Ontario L8S 1C7, Canada; 3 The University of Western Ontario - Roth|McFarlane Hand and Upper Limb Center, St Joseph's Health Center 268 Grosvenor Street, London, Ontario N6A 4L6, Canada; 4 Roth|McFarlane Hand and Upper Limb Centre - Orthopedic Surgery, London, Ontario, Canada

**Keywords:** Distal radius fracture, Patient-rated Wrist Evaluation (PRWE), Prospective cohort, Long term follow-up

## Abstract

**Background:**

Fractures of the distal radius are common. Few studies investigating the extended long term outcomes of participants following a distal radius fracture (especially beyond 2 years) and they have relied on subjective measures or single objective tests to measure participant’s final outcome.

**Objectives:**

The objective of this study was to describe the pain and disability in long-term follow-up of participants after a distal radius fracture. Participants who had previously participated in a prospective study, where baseline and standardized one-year follow-up were performed, were contacted to volunteer to participate in this follow-up (FU) study. Sixty-five participants (17 males, 48 females) with an average age of 57 (SD 13) years at the time of injury and 67 (SD 13 years) at follow-up were evaluated at an average of 11(SD 6) years (range 2-20 years).

**Results:**

The majority of patients (85%) participants reported no change or had less pain and disability (PRWE) (<5 point difference) at their long-term follow-up compared to their one year PRWE scores. One year PRWE scores were found to be predictive (19.1%) of the variability in long term PRWE score (p=0.02). Age, gender, and mechanism of fall were not significant predictors of worsened outcome.

**Conclusion:**

The majority of people that are experiencing no or low patient reported pain and disability one year following a DRF can expect to retain their positive outcome 10-20 years later. This study did not identify how to predict worsened outcome.

## INTRODUCTION

In 1950, Cassebaum *et al.* stated that participants did not experience pain one year after a distal radius fracture (DRF) and that five years later, they would also not have any serious functional complaints [1]. Whereas, Cooney *et al.* stated that participants with distal radius fractures (Colles’ Fracture) have serious complications more frequently than appreciated [2].

Prior to development of patient-reported outcome measures (PRO), DRF outcomes were assessed primarily on the basis of radiological measurements to assess joint incongruity and alignment [2-6]. With the development of PRO, empirical data has shown that the relationship between radiographic and patient-reported outcomes is not strong, especially in older participants. Multiple studies have reported outcomes in prospective cohorts that extend to one year, but the need for longer term is evident [7-11].

Currently, there have been only a few studies investigating the extended long term outcomes of participants following a distal radius fracture (especially beyond 2 years) and they have relied on subjective measures or single objective tests to measure participants final outcome. The primary objective of this study was to determine the mid-to-long term patient-rated pain and disability in participants with a previous distal radius fracture. Specific objectives including evaluating the following over the long term (>1 year) post fracture period:

1. What is the difference between follow-up and 1 year outcome scores in participants following a distal radius fracture? Do participants worsen in the long term?
2. What factors are predictive (radiographic alignment, mechanism of fall, age, sex, length of follow-up and 1-year PRWE) of patient-reported pain and disability in participants with a distal radius fracture?

## MATERIALS AND METHODS

### Participant Requirement

Participants who had previously participated in a prospective study, where baseline and standardized one year follow-up were performed, were contacted to volunteer to participate in this follow-up (FU) study (inclusion criteria: a previous DRF, previous participation in a prospective study (fracture between 1995-2002). Participants were seen by two fellowship-trained hand surgeons at a tertiary-care referral center. Eligible cases agreed to evaluation which involved being sent a package in the mail containing the letter of information, patient-reported outcomes and a return pre-paid postage envelope. All procedures followed were in accordance with the ethical standards of the responsible committee on human experimentation (institutional and national) and with the Helsinki Declaration of 1975, as revised in 2008. Informed consent was obtained from all individual participants included in this study.

#### Independent Variables

Demographic data was obtained for all participants through the previous prospective study and included sex, age, date of fracture, the mechanism of fall (fall on ice or snow, other fall, motor vehicle accident, industrial accident, during sports or other) and attending physician. If values for these variables were missing from the database, original patient records (paper or electronic) were consulted and used to fill in the missing data.

#### Radiographic Parameters

For participants who had a fracture date two to ten years ago, original radiographs could be obtained through the hospital medical imaging data (n=38). Posterior-anterior and lateral radiographs post-reduction (and post definitive treatment) were obtained for each participant. These follow-up radiographs were used to measure radial inclination, ulnar shortening, and volar tilt using a digital goniometer. Fracture type (intra-articular and extra-articular) was also determined from the radiographs. All measurements were performed by a single rater and were according to a standardized process reference A structured review addressing the use of radiographic measures of alignment and the definition of acceptability in patients with distal radius fractures.. The time between injury and post-reduction radiograph was also measured. Dorsal angulation, radial inclination and ulnar variance were examined by a single rater and were measured according to a standardized description of the radiographic parameter (structured review). Overall radiographic alignment was designated as unacceptable (mal-aligned) if the dorsal tilt was >10°, radial inclination <15° and ulnar variance (ulnar positive >3mm) (eRadius International Distal Radius Fracture Study Group: ASSH Specialty Day at AAOS; available at http://www.eradius.com).

#### Patient-reported Outcomes

The primary outcome variable in this study was the follow-up Patient-rated Wrist Evaluation (FU-PRWE). The PRWE is a 15-item patient-reported outcome measure that measures patient’s rated pain and disability and has been shown to be reliable and highly responsive in the distal radius fracture population [12-14]. The PRWE allows participants to rate their levels of wrist pain and disability during a variety of activities of daily living for a total possible score of 100 (0=best possible score, 100=worst possible score). The questionnaire was scored according to the author’s instructions. In 4 cases, the 1-year measure was missing, therefore the 6 month score (PRWE) was carried forward as this is an accepted imputation methods especially given that minimal change between 6 and 12 months has been established [11].

#### Categorizing Outcomes

Follow-up patient-rated pain and disability was measured using the PRWE (FU-PRWE) and compared to 1-year PRWE scores. In order to classify participants as having not improved, having had no change in status or becoming worse, participants were grouped according to the measured difference between their long term follow-up PRWE scores and their 1-year PRWE scores. The standard error of measurement (SEM), measures statistically reliable change, and its value for PRWE (5.22) [15] was used in this study to define whether individual-level change had occurred. Participants were considered to have improved if their FU-PRWE score had decreased 5 or more points, worsened if their FU-PRWE had increased 5, or were considered to have not changed if their score had changed less 5 points.

#### Statistical Analysis

The primary outcome used was FU-PRWE (2-10 years). Descriptive statistics were calculated for all independent variables and the PRWE. Generalized linear models were used to detect differences in PRWE scores over time (repeated factor) between sex current age and length of follow-up between groups categorized as having ‘good’ worse or ‘no change’ 11 years after a distal radius fracture.

Stepwise multiple linear regression models were created to determine factors predictive of outcome. Mechanism of fall, age, sex, length of follow-up and 1-year PRWE scores were included in the model. Statistical significance was set to p<0.05.

Block analysis was also conducted for participants having a fracture 10-20 years ago (n=27) where the same variables were included in the regression. Additional block analyses were conducted examining participants 2-10 years ago (n=38) with the same variables included in addition to operative or non-operative treatment, fracture type (intra/extra articular), post-reduction ulnar variance, post-reduction radial inclination and post-reduction dorsal angulation, acceptable reduction and number of unacceptable parameters was included in the regression model.

## RESULTS

Two-hundred and sixty-two participants who were eligible to participate were contacted and 87 participants agreed to participate in the long term follow-up study. Reasons for refusal included that patients indicated that they were too busy, did not remember that they had a DRF of felt that they were doing well and saw no reason for follow-up. Sixty-five (65) participants completed and returned the questionnaires to the research laboratory (Table **1**). Baseline sex, age, PRWE and one year PRWE scores were calculated for participants that were contacted to participate in this long term follow-up study, but who did not agree/participate. Baseline demographics and one year PRWE scores were similar to those who did participate in this long term follow-up study (Table **2**). Table **3** lists additional patient and fracture characteristics for the participants. The mean age of all participants in the study was 57 (SD 14) years at the time of injury and 67(SD 13) years at follow-up. The range of the current age of participants was 28-85 (60 years). The majority of participants in the cohort were women; with 48 females (74%) and 17 males (26%). The average length of time between injury and follow-up was 11 (SD 6) years. Thirty-eight (38) participants fractured their wrist 2-10 years ago (mid-term outcomes) and twenty-seven (27) participants fractures their wrist 10-20 years ago (long-term outcomes).

### Radiographic Outcomes (n=38, follow-up 2-10 years)

The mean length of time between post-reduction radiographs and the date of fracture was 46 weeks (just under one year). Fig. (**1**) shows a frequency distribution of the date between fracture and follow-up radiographs. Nearly two-thirds (63%) of participants had their follow-up radiographs within the first year following treatment. Twenty-five participants in this cohort of 2-10 years follow-up underwent surgery and eleven had conservative management.

**Fig. (2):**
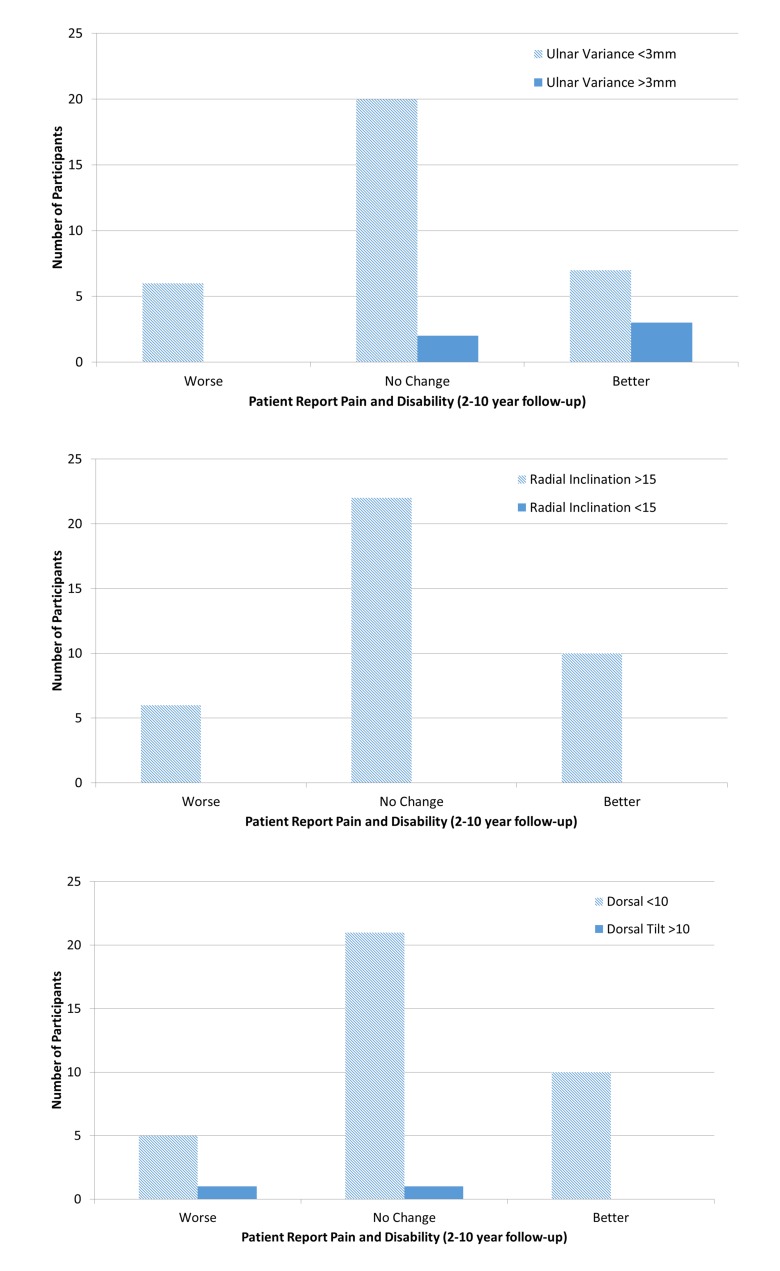
Number of participants with residual mal-alignment (Solid Bars) compared to proper alignment in: A) Ulnar Variance B) Radial Inclination C) Dorsal Tilt.

Twenty-two participants had an intra-articular fracture and 16 had an extra-articular fracture. Seven participants were considered to have residual malunion following their distal radius fracture. Radial inclination was restored in all 38 participants (date of fracture 2-10 years ago). However, 2 participants healed in >10° dorsal angulation (despite having surgery) and 5 participants were ulnar positive (>3mm). Fig. (**2**) shows the number of participants with residual mal-alignment (solid bars) compared to proper alignment in ulnar variance (Fig. **2a**), radial inclination (Fig. **2b**) and dorsal tilt (Fig. **2c**).

### Patient-reported Outcomes

The mean PRWE score for the entire cohort of participants at baseline was 70, at one year 17 and approximately 11 years later 12 (Table **4**).

Long term PRWE scores were compared to 1-year PRWE to categorize participants as having a ‘better’, ‘worse’ or ‘no change’ outcome at long term follow-up. Overall, 55/65 participants (85%) reported having no change or had less patient-reported pain and disability (PRWE) at their long term follow-up (84% 2-10 years, and 85% 10-20 years). Conversely, only 15% of participants had worsened PRWE scores at long term follow-up. Table **5** shows the patient characteristics for each outcome category (number of patients, sex, baseline PRWE, 1-year PRWE and LTFU PRWE, current age, length of follow-up and type of fracture).

### Factors Contributing to Differences in Outcomes

The mean and standard deviation of the baseline, one year and long term follow-up for each of the three outcome groups (better, no change, worse) is shown in Table **6**. When examining baseline measures of patient-reported pain and disability, there were no differences in the baseline of the PRWE scores (p=0.09) between the three groups. There were however differences between the three groups in their 1-year PRWE (p=0.001) and LTFU PRWE scores (p=0.001) (Table **6**). The one year PRWE scores for the ‘improved’ patient cohort was larger (one year PRWE: 33.1 (24.5)) than for the ‘worse” patient cohort (one year PRWE: 12.6 (14.5)). There were no statistical differences detected in the current age of participants between the three groups (worse, no change or better) (p=0.50). As well, there were no statistical differences between groups when examining the length of follow-up (p=0.06), sex (p=0.85).

### Regression Model

#### All Participants (n=65)

One year PRWE predicted 19% of variability in the follow-up scores (adjusted R^2^ value 0.19) (p<0.001) (Table **7a**).

#### Mid-term Follow-up (n=38, 2-10 Years)

One year PRWE predicted should be 43% of variability in the difference between one-year PRWE and FU-PRWE follow-up scores (adjusted R^2^ value 0.42) (p<0.001) (Table **7b**).

#### Long-term Follow-up (n=27, 10-20 Years)

One year PRWE predicted 19% of variability in the mid term follow-up scores (adjusted R2 value 0.159) (p<0.001))Table **8a**).

Table **8b** One year PRWE predicted 51% of variability in the difference between one year and mid term follow-up scores (adjusted R2 value 0.51) (p<0.001).

Table **9b** One year PRWE predicted 20% of variability in the long term follow-up scores (adjusted R2 value 0.) (p<0.001).

## DISCUSSION

This study provided data on a cohort of 65 participants following a distal radius fracture, on average 11 years following their injury. This data may be useful to clinicians and therapists who are interested in determining the long term effects of this frequently occurring upper extremity fracture. The results of this study indicate that the substantial majority of people who incur a DRF who are doing well at one-year will continue to be doing well up to 20 years later; but that 15% of cases who have more pain and disability in the long term cannot be identified on the basis of routine indicators like demographics, fracture type or type of injury. This was a small but long-term cohort that despite the inherent challenges in collecting prospective data over such a prolonged period provided unique insights. This data may be useful to clinicians to tell patients what they can expect in the longer time since post-traumatic arthritis is a concern of patients.

A previous study investigating the factors predictive of patient-reported pain and disability in a cohort of extra-articular distal radius fractures found that injury compensation, education and other co-morbidities explained 16.4% of variance of 1-year PRWE scores and concluded that baseline patient and injury characteristics only played a small role in predicting these 1-score PRWE values [16].

For participants who are having low patient-reported pain and disability, for 85%, they can expect to have low patient-reported pain and disability scores 10 years later (one year PRWE explained 20% of the variance of the term follow-up PRWE scores) and this predictive value was consistent between the three cohorts of 2-20 years, 2-10 years and 10-20 years.

The other baseline values (gender, sex, length of follow-up, energy and mechanism of fall) did not have predictive value for long term follow-up PRWE scores. Potential predictors that could be tested in future studies include radiographic characteristics at the time of injury.

Long-term prospective research has many challenges associated with follow-up, loss of contact information and a potential decrease in participants’ willingness to participate in long term follow-up studies once they have healed. Therefore, there are only a few long term follow-up studies investigating participants following a distal radius fracture [2, 5, 17] Frykman *et al*. in 1967 followed participants having a DRF 3-5 years later and reported subjective symptoms present in 52% of participants (poor outcome in 6%) [17]. Cooney *et al.* in 1980 found that in 565 fractures, 31% of participants had complications such as neuropathies, arthrosis and mal-union [2]. Kopylov *et al*. 1993, in a cohort of 76 participants that 37% of participants described minimal complaints (pain, decreased mobility, cosmetic deformity) approximately 30 years following a distal radius fracture when comparing their fractured and non-fractured wrists, but did find reduced grip strength and decreased wrist flexion when comparing their two wrists [5].

The results of this current study agree with these previous studies investigating longer term outcomes indicating that the majority of participants do not experience poor outcomes in the long term.

A previous study of a cohort of participants measured the PRWE scores for participants with a distal radius fracture at baseline (PRWE score: 75), 8 weeks, 3 months, 6 months and 1 year (PRWE score: 15) and showed that the most change in PRWE scores occurs during the first year [11]. This current long follow-up study also found that the majority of the change in the PRWE score occurs during the first year (Baseline score: 70, one year score: 17). Additionally, as part of the information sent home to the participants in this study, a self-administered co-morbidity questionnaire was given to the participants to complete and send back with the PRWE questionnaire.

Upon closer examination, the 10 participants (in the worsened group), reported having other health problems including high blood pressure, diabetes, depression, osteoarthritis, osteoporosis, rheumatoid arthritis, ulcer or stomach disease as well as other medical problems but these comorbidities were not unique to the ‘worsened’ group.

Future work is needed to examine these co-morbidities more closely and to investigate the relationship between these variables and long term patient-reported pain and disability. Future work is needed to examine these co-morbidities more closely and to investigate the relationship between these variables and long term patient-reported pain and disability.

The patients included in this study represent a minority of those with DRF at our center and thus selection bias must be a concern. Some of the refusals were due to people who could not recall having a DRF. We found our non-respondent demographics to be similar and PRWE scores to be similar suggesting minimal bias.

The strengths of this study included the prospective study design which included actual patient-specific baseline values for the PRWE, our primary outcome measure. Therefore, we were able to avoid recall bias and valid measurement outcomes were consistently used and administered.

This study was not without limitations. This was not an inception prospective study therefore, not all of the participants that were initially included in the prospective study were successfully contacted, available and willing to participate in the long term follow-up study. The low level of participation was expected given the length of follow-up and the low rate of persistent problems after this injury.

People who either did not remember they had a fracture or had no long term effects were not motivated to participate in this study. As well, as this was a long term follow-up study, the clinical management of these distal radius fractures may have changed (treatment guidelines, use of volar or dorsal plates, cast materials) thus affecting the external validity of our findings.

Follow-up radiographic measures of degenerative changes were not measured at long term follow-up. Kopylov *et al.* 1993 found that pain and other complications following a DRF were not attributed to degenerative changes in the distal radioulnar joint, but rather the radiocarpal joint [5]. Post-reduction radiograph measures were not found to be predictive of FU-PRWE in this small cohort of participants (2-10 years).

This may be due to the fact that a very few of these participants had residual mal-reduction and these perturbations in joint alignment were not severe. Future work is needed to isolate patients who are healed in varying degrees of mal-alignment or to recruit patients who would be able to provide follow-up radiographss to examine the effect of mal-alignment and presence of degenerative on the clinical outcomes.

## CONCLUSION

In conclusion, this study provides information regarding participants’ average 11 (2-20) year reported pain and disability following a distal radius fracture in a cohort of 65 participants. Although only 15% of participants had worsened pain and disability 10 years after their DRF, this must be considered in light of the fact that this is a common injury (1/6 of all fractures seen in the emergency room) [18]. Thus, this 15% when applied to the population of DRF represents a large burden in unwanted pain and disability. We did not find baseline predictors of this poor long-term outcome and thus cohorts which are larger and more powered are needed to understand this prognosis.

## Figures and Tables

**Fig. (1):**
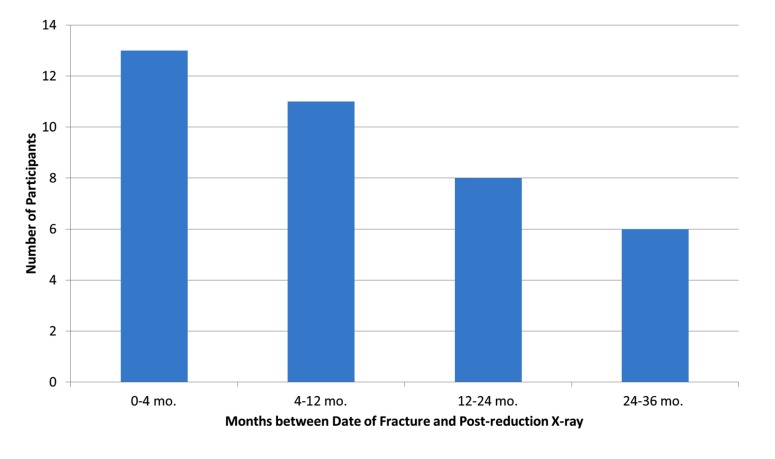
Frequency distribution of the date between fracture and follow-up radiographs.

**Table 1:** Patient recruitment.

	**Contacted**	**Agreed**	**Completed**
**Surgeon 1 (2007-2012)**	51	23	21
**Surgeon 2 (1995-2001)**	93	33	23
**Surgeon 2 (2002-2013)**	118	31	21
**TOTAL**	**262**	**87**	**65**

**Table 2:** Patient characteristics comparing those originally enrolled in the prospective study and those who then participated in the long term follow-up study.

**Characteristic**	**Participated (SD)**	**Did not Participate (SD)**
**Gender**	74% female	75% female
	26% male	25% male
**Age at Fracture**	56.7 (13.8)	54.7 (15.6)
**Baseline PRWE** **One Year PRWE**	69.7 (19.0) 17.4 (21.9)	69.8 (19.0) 16.1 (18.7)
**Characteristic**	**Participated**	**Did not Participate**
**Gender**	74% female	75% female
	26% male	25% male
**Age at Fracture**	56.7 (13.8)	54.7 (15.6)
**Baseline PRWE** **One Year PRWE**	69.7 (19.0) 17.4 (21.9)	69.8 (19.0) 16.1 (18.7)

**Table 3:** Patient and fracture characteristics of patients consented and participated in study.

**Characteristic**	**All Participants**	**2-10 Year Follow-up**	**10-20 Year Follow-up**
**Gender**			
Male	17	6	11
Female	48	32	16
**Age at Fracture (years)**	56.7 (SD 13.8)	61 (SD 13)	50 (SD 13)
**Current Age at Follow-up (years)**	67.3 (SD 12.6)	67 (SD 12)	67 (SD 13)
**Mean Follow-up Length (years)**	10.7 (SD 5.8)	6 (SD 2)	17 (SD 3)
**Mechanism of Fracture**			
1-fall on ice or snow	17	11	6
2-other fall	39	23	16
3-motor vehicle accident	0	0	0
4-industrial accident	0	0	0
5-during sports	0	0	0
6-other	9	4	5

**Table 4:** Overall patient rated wrist evaluation (n=65).

	**Mean (SD)**	**Minimum**	**Maximum**
**Baseline PRWE **	69.7 (19.3)	25	100
**One Year PRWE**	17.4 (21.9)	0	76
**Long Term PRWE**	11.8 (17.7)	0	67.5

**Table 5:** Long term PRWE patient characteristics.

	**All Participants**		**2-10 years**		**10-20 years**	
	**Number of Patients**	**Gender**	**Number of Patients**	**Gender**	**Number of Patients**	**Gender**
**Better PRWE (LTFU)**	24	18 F 6 M	10	8 F 2 M	14	10 F 4 M
**No Change PRWE (LTFU)**	31	22 F 9 M	22	19 F 3 M	9	3 F 6 M
**Worse PRWE (LTFU)**	10	8 F 2 M	6	5 F 1 M	4	3 F 1 M

**Table 6:** PRWE scores categorized by long term outcome (n=65).

	**Baseline PRWE**	**1 Year PRWE**	**LTFU PRWE**
**Better PRWE (LTFU)**	73.1 (15.0)	33.1 (24.5)	9.3 (14.5)
**No Change PRWE (LTFU)**	64.2 (22.2)	6.8 (13.4)	6.5 (13.7)
**Worse PRWE (LTFU)**	75.5 (15.7)	12.6 (14.5)	34.6 (19.6)

**Table 7a:** Model summary of patient characteristics and LTFU-PRWE (All-Participants, n=65).

Model	**R**	R Square	Adjusted R Square	Std. Error of the Estimate		
1	.450^a^	.202	.190		15.9484	

a. Predictors: (Constant), one-year PRWE

**Table:** 

Model		Unstandardized Coefficients		Standardized Coefficients	t	Sig.
		B	Std. Error	Beta		
1	Constant	5.512	2.535		2.174	.033
	1y_prwe	.364	.091	.450	3.995	.000

Dependent Variable: Long Term Follow-up PRWE Score

**Table 7b:** Model summary of patient characteristics and difference between one-year PRWE and FU-PRWE (All-Participants)

Model	**R**	R Square	Adjusted R Square	Std. Error of the Estimate
1	.658^a^	.433	.424	13.22489

a. Predictors: (Constant), 1 year-PRWE

**Table:** 

Model		Unstandardized Coefficients		Standardized Coefficients	t	Sig.
		B	Std. Error	Beta		
1	Constant	3.912	2.102		1.861	.067
	1y_prwe	.523	.076	.658	6.930	.000

Dependent Variable: Difference between Long Term Follow-up and 1-year PRWE Scores

**Table 8a:** Model summary of patient characteristics and LTFU-PRWE (2-10 year follow-up).

Model	**R**	R Square	Adjusted R Square	Std. Error of the Estimate	
1	.447^a^	.200	.176		15.3930	

a. Predictors: (Constant), 1-year PRWE

**Table:** 

Model		Unstandardized Coefficients		Standardized Coefficients	t	Sig.
		B	Std. Error	Beta		
1	Constant	5.953	3.019		1.972	.057
	1y_prwe	.362	.124	.447	2.911	.006

Dependent Variable: Long Term Follow-up PRWE Score

**Table 8b:** Model Summary of Patient Characteristics and Difference between one-year PRWE and FU-PRWE (2-10 year follow-up).

Model	**R**	R Square	Adjusted R Square	Std. Error of the Estimate	
1	.593^a^	.351	.332		13.91523	

a. Predictors: (Constant), 1-year PRWE

**Table:** 

Model		Unstandardized Coefficients		Standardized Coefficients	t	Sig.
		B	Std. Error	Beta		
1	Constant	4.844	2.729		1.775	.085
	1y_prwe	.481	.112	.593	4.291	.000

Dependent Variable: Difference between Long Term Follow-up and 1-year PRWE Scores

**Table 9a:** Model summary of patient characteristics and LTFU-PRWE (10-20 year follow-up).

Model	**R**	R Square	Adjusted R Square	Std. Error of the Estimate
1	.437^a^		.191	17.7505

a. Predictors: (Constant), 1-year PRWE

**Table:** 

Model		Unstandardized Coefficients		Standardized Coefficients	t	Sig.
		B	Std. Error	Beta		
1	Constant	5.073	5.028		1.009	.323
	1y_prwe	.377	.155	.437	2.430	.023

Dependent Variable: Long Term Follow-up PRWE Score

**Table 9b:** Model summary of patient characteristics and difference between one-year PRWE and FU-PRWE (10-20 year follow-up).

Model	**R**	R Square	Adjusted R Square	Std. Error of the Estimate
1	.717^a^	.514	.494	13.51813
2	.770^b^	.593	.559	12.62200

a. Predictors: (Constant), 1-year PRWE b. Predictors: (Constant), 1-year PRWE, mechanism_fall

**Table:** 

Model		Unstandardized Coefficients		Standardized Coefficients	t	Sig.
		B	Std. Error	Beta		
1	Constant	.659	3.829		.172	.865
	1y_prwe	.607	.118	.717	5.137	.000
2	Constant	7.403	4.744		1.560	.13
	1y_prwe	.655	.113	.774	5.825	0.000
	mechanism)_fall	-3.140	1.452	-.288	-2.162	0.041

Dependent Variable: Difference between Long Term Follow-up and 1-year PRWE Scores

## References

[1] Cassebaum W.H. (1950). Colles’ fracture; a study of end results.. J. Am. Med. Assoc..

[2] Cooney W.P., Dobyns J.H., Linscheid R.L. (1980). Complications of Colles’ fractures.. J. Bone Joint Surg. Am..

[3] McQueen M., Caspers J. (1988). Colles fracture: does the anatomical result affect the final function?. J. Bone Joint Surg. Br..

[4] Cai L., Zhu S., Du S., Lin W., Wang T., Lu D., Chen H. (2015). The relationship between radiographic parameters and clinical outcome of distal radius fractures in elderly patients.. Orthop. Traumatol. Surg. Res..

[5] Kopylov P., Johnell O., Redlund-Johnell I., Bengner U. (1993). Fractures of the distal end of the radius in young adults: a 30-year follow-up.. J. Hand Surg. [Br.].

[6] Goldfarb C.A., Rudzki J.R., Catalano L.W., Hughes M., Borrelli J. (2006). Fifteen-year outcome of displaced intra-articular fractures of the distal radius.. J. Hand Surg. Am..

[7] Constand M.K., MacDermid J.C., Law M., Dal Bello-Haas V. (2014). Patient-centered care and distal radius fracture outcomes: a prospective cohort study analysis.. J. Hand Ther..

[8] Grewal R., MacDermid J.C. (2007). The risk of adverse outcomes in extra-articular distal radius fractures is increased with malalignment in patients of all ages but mitigated in older patients.. J. Hand Surg. Am..

[9] MacDermid J.C., Donner A., Richards R.S., Roth J.H. (2002). Patient versus injury factors as predictors of pain and disability six months after a distal radius fracture.. J. Clin. Epidemiol..

[10] Anzarut A., Johnson J.A., Rowe B.H., Lambert R.G., Blitz S., Majumdar S.R. (2004). Radiologic and patient-reported functional outcomes in an elderly cohort with conservatively treated distal radius fractures.. J. Hand Surg. Am..

[11] MacDermid J.C., Richards R.S., Roth J.H. (2001). Distal radius fracture: a prospective outcome study of 275 patients.. J. Hand Ther..

[12] MacDermid J.C. (1996). Development of a scale for patient rating of wrist pain and disability.. J. Hand Ther..

[13] MacDermid J.C., Turgeon T., Richards R.S., Beadle M., Roth J.H. (1998). Patient rating of wrist pain and disability: a reliable and valid measurement tool.. J. Orthop. Trauma.

[14] MacDermid J.C., Richards R.S., Donner A., Bellamy N., Roth J.H. (2000). Responsiveness of the short form-36, disability of the arm, shoulder, and hand questionnaire, patient-rated wrist evaluation, and physical impairment measurements in evaluating recovery after a distal radius fracture.. J. Hand Surg. Am..

[15] Schmitt J.S., Di Fabio R.P. (2004). Reliable change and minimum important difference (MID) proportions facilitated group responsiveness comparisons using individual threshold criteria.. J. Clin. Epidemiol..

[16] Grewal R., MacDermid J.C., Pope J., Chesworth B.M. (2007). Baseline predictors of pain and disability one year following extra-articular distal radius fractures.. Hand (NY).

[17] Frykman G. (1967). Fracture of the distal radius including sequelae--shoulder-hand-finger syndrome, disturbance in the distal radio-ulnar joint and impairment of nerve function. A clinical and experimental study.. Acta Orthop Scand.

[18] Owen R.A., Melton L.J., Johnson K.A., Ilstrup D.M., Riggs B.L. (1982). Incidence of Colles’ fracture in a North American community.. Am. J. Public Health.

